# Differences in SOM Decomposition and Temperature Sensitivity among Soil Aggregate Size Classes in a Temperate Grasslands

**DOI:** 10.1371/journal.pone.0117033

**Published:** 2015-02-18

**Authors:** Qing Wang, Dan Wang, Xuefa Wen, Guirui Yu, Nianpeng He, Rongfu Wang

**Affiliations:** 1 Resources and Environment College, Anhui Agricultural University, Hefei, China; 2 Key Laboratory of Ecosystem Network Observation and Modeling, Institute of Geographic Sciences and Natural Resources Research, Chinese Academy of Sciences, Beijing, China; Chinese Academy of Sciences, CHINA

## Abstract

The principle of enzyme kinetics suggests that the temperature sensitivity (*Q*_10_) of soil organic matter (SOM) decomposition is inversely related to organic carbon (C) quality, i.e., the C quality-temperature (CQT) hypothesis. We tested this hypothesis by performing laboratory incubation experiments with bulk soil, macroaggregates (MA, 250–2000 μm), microaggregates (MI, 53–250 μm), and mineral fractions (MF, <53 μm) collected from an Inner Mongolian temperate grassland. The results showed that temperature and aggregate size significantly affected on SOM decomposition, with notable interactive effects (*P*<0.0001). For 2 weeks, the decomposition rates of bulk soil and soil aggregates increased with increasing incubation temperature in the following order: MA>MF>bulk soil >MI(*P* <0.05). The *Q*_10_ values were highest for MA, followed (in decreasing order) by bulk soil, MF, and MI. Similarly, the activation energies (*E_a_*) for MA, bulk soil, MF, and MI were 48.47, 33.26, 27.01, and 23.18 KJ mol^−1^, respectively. The observed significant negative correlations between *Q*_10_ and C quality index in bulk soil and soil aggregates (*P*<0.05) suggested that the CQT hypothesis is applicable to soil aggregates. Cumulative C emission differed significantly among aggregate size classes (*P* <0.0001), with the largest values occurring in MA (1101 μg g^−1^), followed by MF (976 μg g^−1^) and MI (879 μg g^−1^). These findings suggest that feedback from SOM decomposition in response to changing temperature is closely associated withsoil aggregation and highlights the complex responses of ecosystem C budgets to future warming scenarios.

## Introduction

Soil aggregates play important roles in maintaining soil structure, fertility, and stability, and in influencing the decomposition dynamics of soil organic matter (SOM) [1–3]. Soil aggregates store large quantities of SOM [1,2], and therefore the distribution of SOM within aggregates is an important factor for its turnover [4]. Temperature is one of the most important factors influencing SOM decomposition, and is considered to be positively correlated with the decomposition rate of SOM [3]. Under global warming scenarios, the response of SOM decomposition in different soil aggregates to temperature change is unquestionably important for predicting the global pattern and magnitude of soil carbon (C) storage in the future [5].

The temperature sensitivity (*Q*_10_) of SOM decompositioncan be depicted by exponential models [6,7] and Arrhenius models [8,9]. Exponential models better reflect the effects of temperature on SOM decomposition, whereas Arrhenius models provide mechanistic explanations for SOM decomposition and *Q*_10_. Recently, there has been a common assertion that the composition of SOM can affect the temperature sensitivity of SOM decomposition; however, experimental and model evidence havenot been presented. Some studies have included a series of incubation experiments to investigate the effect of soil aggregation on SOM decomposition, but the results were inconsistent. Manna *et al*. demonstrated that macroaggregates (250–2000 μm) were dominant in the decomposition process of SOM [10]; however, other studies have indicated that microaggregates (53–250μm) [11] or mineral fractions (<53 μm)[12] are more important. Understanding the differences in SOM decomposition and its temperature sensitivity in different soil aggregates is therefore essential to accurately assess the effects of future warming on soil C storage.

The principle of enzyme kinetics suggests that the *Q*_10_ value of SOM decomposition is controlled by the C quality of substrates utilized by microorganisms [8], and that a higher activation energy (*E*_*a*_) is required to mineralize low-quality C substrates (C quality-temperature [CQT] hypothesis) [13,14].The CQT hypothesis have been confirmed by the data of soil incubation experiments [14–16].However, the CQT hypothesis has not been experimentally tested for different soil aggregates with different C qualities resulting from different physical and chemical protective mechanisms [12,17,18].

In this study, incubation experiments were performed using soils oftypical temperate grassland in Inner Mongolia, to assess the SOM decomposition and temperature sensitivity in different-sized aggregates (macroaggregates, microaggregates, and mineral fractions) and to test the CQT hypothesis. The main objectives of this study were to investigate (i) how decomposition of SOM varies among aggregate size and temperature; (ii) how *Q*_10_ values differ among soil aggregates; and (iii) whether CQT hypothesis is appropriate for soil aggregates.

## Materials and Methods

### Study sites

Soil samples were collected froma typical temperate grassland at the Inner Mongolia Grassland Ecosystem Research Station (IMGERS), Chinese Academy of Sciences (43°33′17.33″N, 116°40′32.44″E). This region is characterized by a semi-arid continental climate with mean annual precipitation of 345 mm and mean annual temperature of 1.1°C [19]. The soil is classified as Calcic Chernozem with loamy sand texture. The experimental plot was established at IMGERS in 1999 by fencing off a section of grassland that was previously open to free grazing by sheep and cattle. The predominant vegetation consists of grasses, including *Leymus chinensis*, *Stipa grandis*, and *Cleistogenes squarrosa* [19].

### Sampling and pretreatment

Soil samples (0–20 cm depth layer) were randomly collected from 10 locations in the long-term experimental plot. In the laboratory, we manually removed roots and visible organic debris from the samples. After sieving (<2-mm mesh), approximately 100 g of soil from each sample was air-dried for analysis of basal properties, including C content, nitrogen (N) content, and pH. The C and N contents of bulk soil and aggregates were measured using an elemental analyzer (Elementarvario max, Germany). Soil pH was determined using a pH meter (Mettler Toledo Delta 320, Switzerland) in a slurry of soil and distilled water (1:2.5)

The soil water holding capacity (WHC, %) of the soil and aggregates were measured by oven drying.The remaining soil was stored at 4°C. All samples were pre-sieved (2 mm diameter) prior to wet-sieving to remove stones and coarse organic matter and todefine the initial dimensions of the aggregates for analysis.Water-stable aggregateswere separated using two sieves similar in principle to a Yoder wet-sieving apparatus. The apparatus was modified to handle stackedsieves and to enable complete recovery of all particle fractions from individual samples [20]. Fresh soils were shaken for 2 min in two sieves (250 and 53 μm diameter) to generate three aggregate fractions: macroaggregates (MA, 250–2000 μm), microaggregates (MI, 53–250 μm), and mineral fractions (MF, <53 μm) [2].

### Experimental design

Two incubation experiments were performed.

Experiment I was designed to investigate the difference in the temperature sensitivity of SOM decomposition among soil aggregates. In brief, 40-g samples of fresh bulk soil or aggregates, adjusted to 60% WHC, were placed into incubation bottles (5 cm diameter, 10cmheight) and mixed with 10 g of quartz sand. The soil samples were then pre-incubated at 20°C and constant humidity (80%) for 1 week. The samples were then incubated at different temperatures (5, 10, 15, 20, and 25°C) for 2 weeks (*n* = 3; for each temperature treatment), and SOM decomposition rates were measuredsix times, at 0, 1, 3, 5, 7, and 14 d.

Experiment II was designed to examine differences in decomposition over long-term incubation among aggregates. Soil samples (40 g of fresh soils or aggregates, adjusted to 60% WHC and mixed with 10 g of quartz sand) were pre-incubated at 20°C and 80% humidity for 1 week and then placed into incubation bottles (25°C). During the 169-d incubation, the SOM decomposition rate at 25°C was measured 14 times, on days 0, 1, 3, 5, 7, 14, 21, 28, 35, 42, 49, 56, 84, 112, 140, and 169.

SOM decomposition rates were measured using an automatic system, which was modified from the continuous gas flow system of Cheng and Virginia [21]. This system consisted of a Li-COR CO_2_ analyzer (Li-7000), an electric water bath to control incubation temperature, an air-flow controller, soda-line equipment to control the initial CO_2_ concentration, an auto-sampler on a turn-plate, automatic transformation valves to control the sample bottle, and a data collector, which was the same as the equipment of He *et al*. [22].

### Calculation method

The SOM decomposition rates was calculated from the slope of the CO_2_ concentration and conversion factors as follows:

$$R=\frac{C\times V\times \alpha \times \beta }{m}$$ (1)

where *R* is SOM decomposition rate (μg Cg^−1^h^−1^); *C* is the slope of the change in CO_2_ concentration; *V* is the volume of the incubation bottle and gas tube; *m* is the soil weight (g); *α* is the conversion coefficient for CO_2_ mass; and *β* is a conversion coefficient of time.

The *Q*_10_ of SOM decomposition was calculated using the following exponential equations [23]:

$$R=A\times e^{bT}$$ (2)

$$Q_{10}=e^{10b}$$ (3)

where *R* is SOM decomposition rate (μg C g^−1^ h^−1^), *T* is temperature (°C), and A and b are the exponential fit parameters that describe the intercept and slope of the line, respectively.

According to the Arrhenius equation, *SR* is a function of a pre-exponential parameter (A), the activation energy (*Ea*), the gas constant R and temperature (*T*)(Eq. 4). Eq. 5 was used toassess the relationship between *Ea* and *Q*_*10*_, derivedfrom Eq. 4 [23].

$$R=A\times e^{-E_{a}/RT}$$ (4)

$$E_{a}=R\times Ln(Q_{10})/(\frac{1}{T_{1}}-\frac{1}{T_{2}})$$ (5)

where R is the gas constant (8.314 J mol^−1^) and *T*_*1*_ and *T*_*2*_ are temperatures (*K*) indicating the 10°C temperature range for the corresponding *Q*_10_ (i.e., *T*_*1*_ + 10 = *T*_*2*_). In calculating *E*_*a*_, it was assumed that *Q*_10_ represented the range *T_-5_* to *T*_*+5*_, where T was the average incubation temperature.

### Statistical analysis

One-way ANOVA and the least significant difference method (LSD) were used to explore the effects of aggregate size, incubation temperature, and their interactions on SOM decomposition.Regression analyses were used to evaluate the relationships between the C quality index and *Q*_10_ and *E*_*a*_. Differences were considered significant at *P*<0.05. All statistical analyses were conducted using SPSS 13.0 for windows (SPSS Inc., Chicago, IL, USA).

## Results

### Soil properties

The C and N content and C:N ratios differed significantly (*P* <0.0001) among bulk soil and aggregates (Table 1). The highest C content was observed in MA, and the lowest in bulk soil and MI; the similar trends were also observed for N content. The C:N ratio increased with increasing aggregate size.

**Table 1:** Carbon and nitrogen content of different soil fractions in Inner Mongolian grasslands.

	Total carbon content(%)	Total nitrogen content(%)	C:N ratio
Bulk soil	1.855 (0.013)^c^	0.175 (0.007)^b^	10.611 (0.356)^b^
MA: 250–2000 μm	2.089 (0.027)^b^	0.182 (0.013)^b^	11.546 (0.774)^a^
MI: 53–250 μm	1.383 (0.006)^d^	0.148 (0.008)^c^	9.343 (0.502)^c^
MF: <53 μm	2.268 (0.011)^a^	0.247 (0.016)^a^	9.200 (0.625)^c^
*F*	1986.467	49.256	15.316
*P*	<0.0001	<0.0001	<0.0001

MA, macroaggregates; MI, microaggregates; MF, mineral fraction.Data are means (SD) (*n* = 3); data with different superscript letters within a column are significantly different at *P*<0.05.

### Decomposition rate and temperature sensitivity

**Fig 1:**
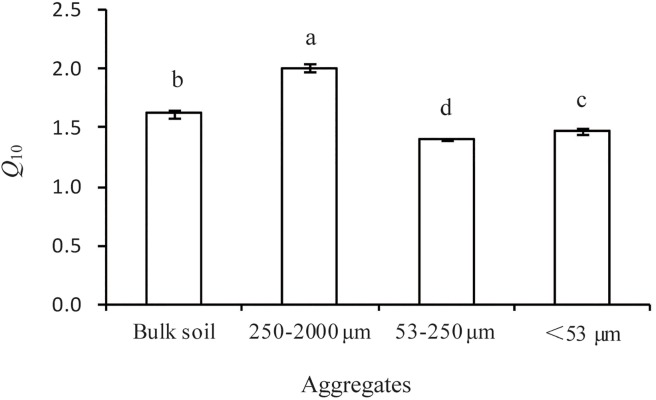
Differences in the temperature sensitivity (*Q*_10_) of SOM decomposition in different aggregate size fractions. The *Q*_10_ values were calculated using an exponential equation. Values indicate the mean (*n* = 3); bars indicate the SD.Different letters indicate significant differences at *P*<0.05.

**Fig 2:**
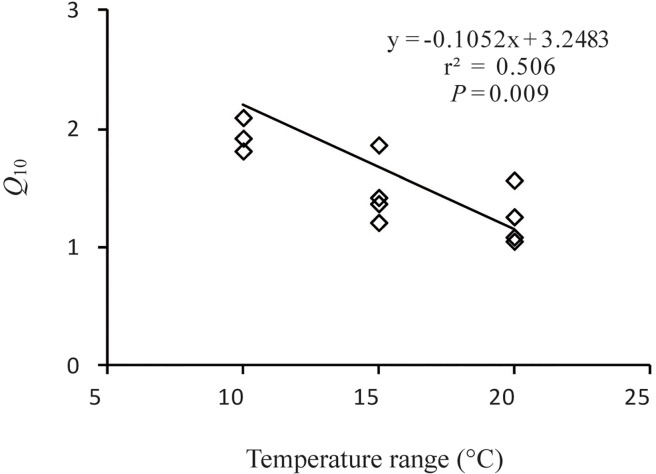
Relationship between temperature sensitivity (*Q*_10_) and incubation temperature. The *Q*_10_ values were calculated using the Arrhenius equation.

**Fig 3:**
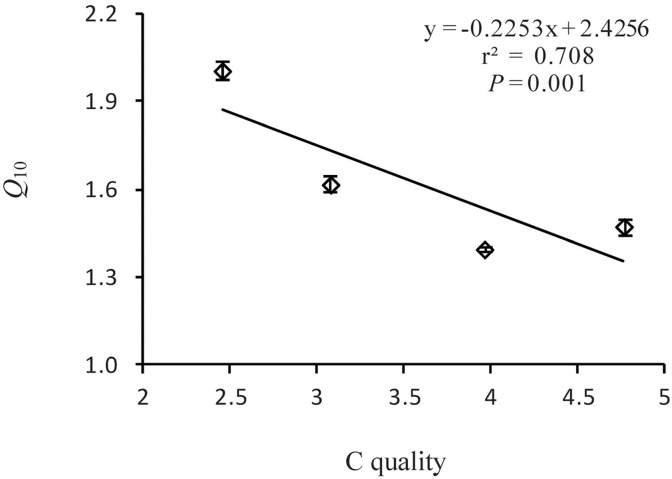
Relationship between temperature sensitivity (*Q*_10_) and SOC quality. The *Q*_10_ values werecalculated using an exponential equation. Values were the means (*n* = 3); bars indicate the SD.

Temperature and aggregate size had a significant influence on SOM decomposition rates, with notable interactive effects (*P*<0.0001). In addition, the *Q*_10_ values of SOM decomposition calculated using either the exponential model orthe Arrhenius model differed significantly among aggregate sizes (Fig. 1, S1 Fig., Table 2), ordered as follows: MA > MF > bulk soil > MI. We also found that the differences in *Q*_10_ values decreased with increasing temperature (F = 299.98, *P*<0.0001) (Fig. 2,Table 3). Significant negative correlations were observedbetween *Q*_10_ and the C quality index (*P* = 0.001, Fig. 3, S2 Fig.). Similarly, negative correlations were observed between *Q*_10_ values and *Ea* (F = 374.43, *P*<0.0001, Table 3), which is consistent with the CQT hypothesis.

**Table 2:** Parameters for the empirical exponential equation of SOM decomposition.

	*A*(mgkg^-1^d^-1^)	*b*	R^2^	*Q*_10_
Bulk soil	3.071 (0.101)	0.048 (0.002)	0.910	1.618 (0.028)^b^
MA, 250–2000 μm	2.450 (0.070)	0.070 (0.002)	0.879	2.001 (0.031)^a^
MI, 53–250 μm	3.957 (0.042)	0.033 (0.001)	0.789	1.400 (0.008)^d^
MF, <53 μm	4.759 (0.094)	0.039 (0.002)	0.806	1.474 (0.026)^c^
*F*				354.698
*P*				<0.0001

MA, macroaggregates; MI, microaggregates; MF, mineral fraction. Data are means (SD) (*n* = 3); data with different superscript letters within a column are significantly different at *P*<0.05. A and b are the exponential fit parameters describing theintercept and slope, respectively. *Q*_10_ is the temperature sensitivity of SOM decomposition.

**Table 3:** Temperature sensitivity (*Q*_10_) and activation energy (*E*_*a*_) for different aggregate size classes calculated using the Arrhenius equation.

	Temperature sensitivity (*Q*_10_)				Activation energy (*E*_*a*_)
	5–15°C	10–20°C	15–25°C	Mean (SD)	
Bulk soil	1.815 (0.128)^b^	1.372 (0.068)^bc^	1.569 (0.062)^a^	1.586 (0.032)^b^	33.258 (1.205)^b^
MA, 250–2000 μm	3.349 (0.309)^a^	1.866 (0.144)^a^	1.261 (0.062)^b^	2.169 (0.053)^a^	48.467 (0.979)^a^
MI, 53–250 μm	1.923 (0.017)^b^	1.214 (0.053)^c^	1.090 (0.046)^c^	1.409 (0.007)^c^	23.178 (0.399)^d^
MF, <53 μm	2.098 (0.012)^b^	1.424 (0.041)^b^	1.055 (0.033)^c^	1.526 (0.023)^b^	27.009 (1.184)^c^
*F*	54.047	31.200	61. 375	299.976	374.432
*P*	<0.0001	<0.0001	<0.0001	<0.0001	<0.0001

MA, macroaggregates; MI, microaggregates; MF, mineral fraction.Data are means (SD) (*n* = 3); data with different superscript letters within a column are significantly different at *P*<0.05.

### Cumulative C emission

**Fig 4:**
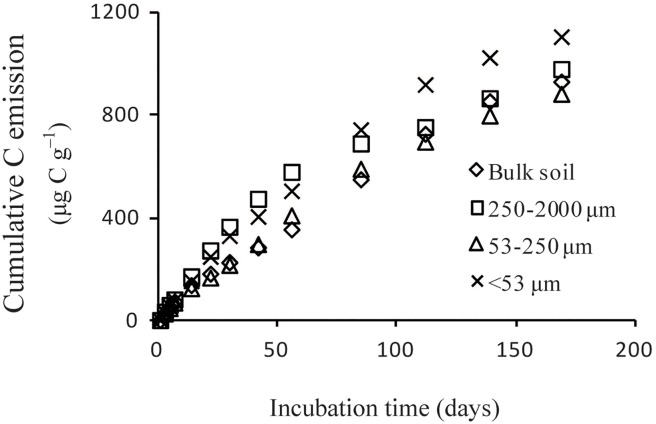
Cumulative C emission in different aggregatesize classes during incubation at 25°C.

Aggregate size significantly influenced cumulative C emission (F = 269.89, *P*<0.0001), with the highest values occurring in MA and the lowest in MI (Fig. 4). Differences in cumulative C emission increased with increasing incubation time, althoughthe decomposition rate decreased for all soil aggregates.

## Discussion

### Decomposition of soil aggregates

Aggregate size had a significant influence on SOM decomposition. However, the decomposition rate of SOM still varied among soil aggregates.Some studies have found that SOM decomposition rates generally decrease with decreasing aggregate size [3,24], and that MA contains a greater proportion of decomposable C than whole soil [25]. However, others have reported that MI is a higher decomposition rate than MA[11,26]. The present results show that MA contains more organic C than MI (Table 1), which supports the concept of aggregate size classes [27,28]. Differences in organic C content among soil aggregates may result in the variability of cumulative C emission. One plausible explanation is that particulate organic matter in MA can decompose into MI, which contains less organic C [17]. Moreover, the soil C:N ratio of the aggregates, as one of the indicators of the degradability of SOM, increases with increasing aggregate size [29], which resultsin the higher cumulative C emission in MA [30]. However, some studies have demonstrated that soils with lower C:N ratios generally have higher CO_2_ production up to a certain threshold [23,31].

Our findings show that C emissions are derived mainly from MA during relatively short-term incubation (56 d) and from MF during longer-term (more than 56 d) incubation, which is consistent with the findings of Christensen [32]. Dou *et al*. demonstrated that the condensation and molecular complexity of humic acids decrease with increasing aggregate size, accompanied by an increase in activation grade [33]. Although recent studies have suggested that recalcitrant molecules (e.g., lignin) contribute minimally to the long-term stability of SOM, except for somechar [34–36], the substrate molecular structure remains a critical factor during the early stages of SOM decomposition where the substrate isaccessible to microbes. Moreover, the soil microbial biomass varies among aggregates of different particle sizes, with greater values generally observed in MA [37].

### Temperature sensitivity related to aggregate size

Aggregate size had a significant effect on the temperature sensitivity of SOM decomposition. The most widelymodels for different SOM pools use fixed *Q*_10_ values of 1.5–2 [38–42]. In the present study, the *Q*_10_ values ranged from 1.4 to 2.2, which is similar to the results of previous incubation experiments [43–46]. On the basis of thermodynamic principle, the decomposition rates of SOM in different aggregates were more sensitive to lower temperature than to higher temperature [47]. Here, the maximum *Q*_10_ value was observed for MA, from 5 to 15°C. In contrast, Tan *et al*. reported that the maximum *Q*_10_ value occurred in MI [3]. These inconsistent results indicate that complex mechanisms control the temperature sensitivity of SOM decomposition, which depended on organic-mineral interactions [48–50], the quality and structure of the SOM [15,49], and other environmental factors, e.g., moisture [51].

Our findings show that MA and MF have higher *Q*_10_, implying that they will be more sensitive to future climate warming and could generate a positive feedback for global warming. The variability in *Q*_10_ and its dependence on aggregate size have important implications for regional and global ecosystem C modeling, specifically for predicting the response of terrestrial ecosystems to future global warming. Experiments have been conducted experiments toexplore the temperature sensitivity of the SOM decomposition of different aggregates and its underlying mechanisms, including substrate availability [6], physical and chemical protection [32], and microbial activity [52]. Craine *et al*. demonstrated that C quality was particularly important for the temperature sensitivity of SOM decomposition [41]. Inconsistent findings have been reported, including increases [53,54], no change [5,39], or decreases [55,56] in the *Q*_10_ of SOM decomposition with increasing C quality. In the future, it will therefore be essential to investigate the important physical and chemical factors controlling SOM decomposition in different aggregates.

Similarlyto other studies [41,57–59], we also observed that *Q*_10_ and *E*_*a*_ were both inversely related to this index. These findings are consistent with the CQT hypothesis [55], which proposes that the decomposition of low-quality SOM requires higher *E*_*a*_ and is more sensitive to temperature than the degradation of high-quality SOM [9,59]. The present results provide new evidence that the CQT hypothesis is applicable to soil aggregates.The robust CQT relationship [41] deserves more detailed examination, because it may be used to improve models for predicting the SOM response to anticipatedwarming. As proposed by Wagai *et al*. [49], some issues for better understanding the CQT hypothesis need to be addressed in the future by (i) providing a better definition of ‘C quality’ based on the actualmolecular structure of organic compounds in soil; (ii) distinguishing the active or microbial accessible fraction from bulk SOM; and (iii) simultaneously assessing the temperature effect on easily soluble C pools and microbial biomass C in addition to microbial respiration.

## Conclusion

The decomposition rates of SOM and the corresponding *Q*_10_ values differed remarkably among aggregate sizes, and both increased with aggregate size. *Q*_10_ and *Ea* were inversely correlated with organic C quality in different soil aggregates, supporting the CQT hypothesis with respect to soil aggregate classes. Therefore, future research on the relationship between SOM decomposition and C quality should consider not only C quality with different incubation times and different SOM, but also soil physical propertiesto explore internal mechanisms controlling the decomposition rates of SOM in aggregates, and their temperature sensitivity. Moreover, differences in SOM decomposition rates and temperature sensitivity are important and should be incorporated into models to improve prediction in the future.

## Supporting Information

S1 Fig Energy of activation (*E*_*a*_) of SOM decomposition for different aggregate size fractions. Values are the mean (*n* = 3); bars indicate the SD. Different letters indicate a significant differences at *P*<0.05.
(TIF)

S2 Fig Relationship between energy of activation (*E*_*a*_) and SOC quality. *E*_*a*_ was calculated by the Arrhenius equation and SOC quality was calculated by the exponential equation. Values are the mean (*n* = 3); bars indicate the SD.
(TIF)

S1 File Data of SOM decomposition rates for PONE-D-14-18377. (XLSX)
