# Supplementary figures and images for: Differences in SOM Decomposition and Temperature Sensitivity among Soil Aggregate Size Classes in a Temperate Grasslands
Source: PLoS One. 2015 Feb 18;10(2):e0117033. doi: 10.1371/journal.pone.0117033 (PMC4334239; doi:10.1371/journal.pone.0117033)

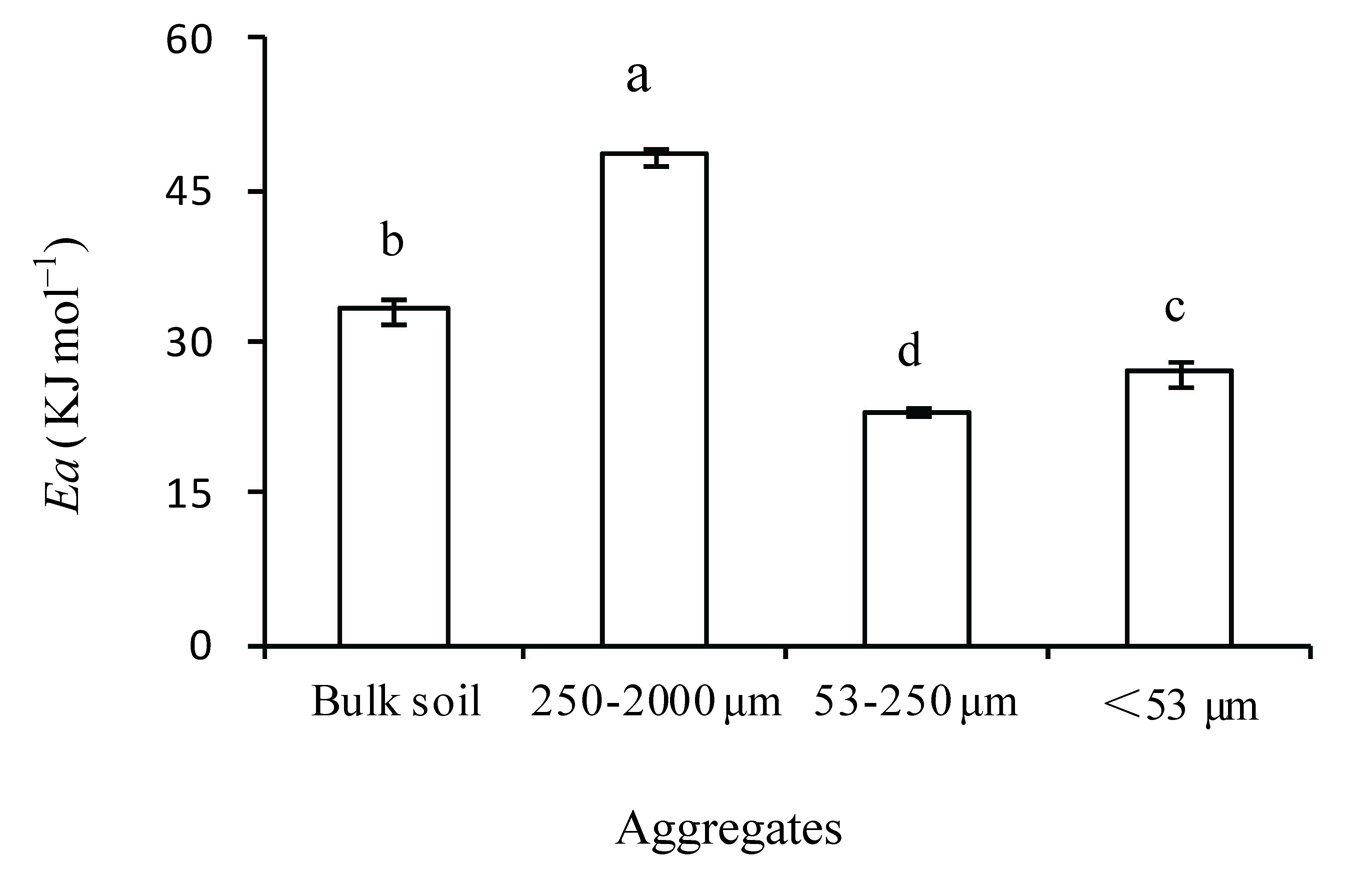

Supplement: S1 Fig — Values are the mean (n = 3); bars indicate the SD. Different letters indicate a significant differences at P<0.05. (TIF) [file pone.0117033.s001.tif]

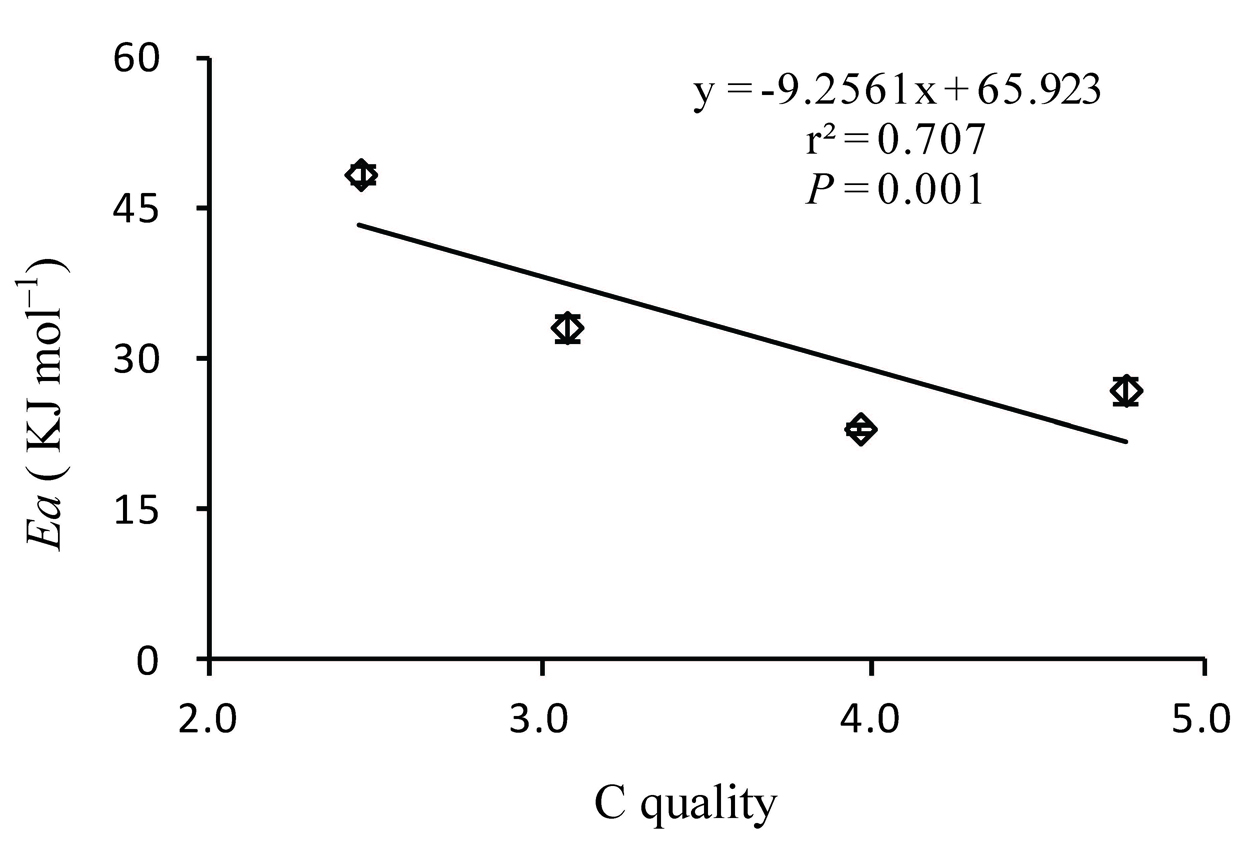

Supplement: S2 Fig — E a was calculated by the Arrhenius equation and SOC quality was calculated by the exponential equation. Values are the mean (n = 3); bars indicate the SD. (TIF) [file pone.0117033.s002.tif]
